# Damage Detection and Identification on Elevator Systems Using Deep Learning Algorithms and Multibody Dynamics Models

**DOI:** 10.3390/s25010101

**Published:** 2024-12-27

**Authors:** Josef Koutsoupakis, Dimitrios Giagopoulos, Panagiotis Seventekidis, Georgios Karyofyllas, Amalia Giannakoula

**Affiliations:** 1 School of Mechanical Engineering, Aristotle University of Thessaloniki, 54124 Thessaloniki, Greece; jkoutsoup@meng.auth.gr (J.K.); seventp@meng.auth.gr (P.S.); gdkaryof@meng.auth.gr (G.K.); 2 KLEEMANN Group, 61100 Kilkis, Greece; a.giannakoula@kleemannlifts.gr

**Keywords:** structural healthy monitoring, deep learning, multibody dynamics, signal analysis, data acquisition

## Abstract

Timely damage detection on a mechanical system can prevent the appearance of catastrophic damage in it, as well as allow for better scheduling of its maintenance and repair process. For this purpose, multiple signal analysis methods have been developed to help identify anomalies in a system, through quantities such as vibrations or deformations in its critical components. In most applications, however, these data may be scarce or inexistent, hindering the overall process. For this purpose, a novel approach for damage detection and identification on elevator systems is developed in this work, where vibration data obtained through physical measurements and high-fidelity multibody dynamics models are combined with deep learning algorithms. High-quality training data are first generated through multibody dynamics simulations and are then combined with healthy state vibration measurements to train an ensemble of autoencoders and convolutional neural networks for damage detection and classification. A dedicated data acquisition system is then developed and integrated with an elevator cabin, allowing for condition monitoring through this novel methodology. The results indicate that the developed framework can accurately identify damages in the system, hinting at its potential as a powerful structural health monitoring tool for such applications, where manual damage localization would otherwise be considerably time-consuming.

## 1. Introduction

The increase in the population of urban areas as well as the need for building structures in a limited area has led to the construction of a large number of high-rise buildings, especially near city centers or industrial areas, where there are high demands for occupation or office space and working environments. This high number of floors in said buildings makes the use of elevators virtually mandatory, as scaling up and down between multiple floors would require a prohibiting amount of time or even effort. To this end, elevator systems have been constantly developed and improved, aiming to make rides between floors smoother, faster, and most importantly, safer. Additionally, analysis of the wear in an elevator’s critical subsystems, such as brakes, steel wires, counterweight, and cabin [1,2,3,4,5,6,7,8,9,10,11] has led to the development of a variety of methods for detecting anomalies in the system, based on sensor data such as vibrations, strains, power, etc., aiming to ensure the timely detection of damages in the system as well as its proper maintenance.

The rapid increase in Machine Learning (ML) and Deep Learning (DL) algorithms has driven many researchers to develop novel frameworks for ML/DL-based Structural Health Monitoring (SHM) of mechanical systems, using algorithms such as Convolutional Neural Networks (CNNs) [3,4,5,6,7,12,13], Multilayer Perceptrons (MLPs) [9], Graph Neural Networks (GNNs) [14] and even combinations of multiple methods, through decision fusion [8]. These methods, being data-driven, are directly related to the availability of training data, especially when supervised learning methods are employed, as they require labeled datasets from every healthy state of interest. This is because damaged state data are usually scarce or even inexistent since the operation of a system usually stops when damage is detected in it, aiming to avoid the propagation of damage in it and prevent potentially harmful accidents. Damaged state data could potentially be produced by inflicting artificial damage to the system, but this is usually impossible due to the prohibiting costs of deliberately damaging a structure. Due to the abovementioned limitations, ML/DL-based SHM applications are usually restricted to experimental cases in laboratory environments or anomaly detection applications, where healthy state data alone are sufficient.

Aiming to mitigate this shortcoming of purely data-driven methods, numerical models, such as Finite Element (FE) or Multibody Dynamics (MBD), have been explored as a means of labeled data generation. By increasing the fidelity of these numerical models through powerful optimization algorithms [15], labeled training data can be generated through simulations of FE [16] or MBD [17,18,19] for all health states of a system, while the number of samples is also virtually unlimited, as the only relevant cost of simulating a particular health state is that of processing time and computational resources. These methods have been proven successful in SHM of mechanical systems, albeit, in laboratory conditions, with applications including even subtle damages to subsystems such as the sliding doors of an elevator system [17]. While other approaches, such as Generative Adversarial Networks (GANs) [20,21] or transfer learning methods [22], can be used for generating additional datasets in order to deal with data imbalance, thus leading to improved prediction accuracy, these methods require at least a small number of damaged state datasets for their implementation. The approach of using numerical models to bridge this data-induced gap, while potentially not achieving the same levels of accuracy, can be implemented even in cases where damaged state data are considered unavailable.

Timely detection of damages, however, requires that the system be monitored constantly during operation and, as such, online Data Acquisition (DAQ) is required to detect changes in its response which may be related to the appearance of faults in its subsystems. To achieve optimal maintenance of the system, it is also crucial that this detection takes place while a fault is in its early stages before it can expand and propagate to other parts of the structure. To this end, a health monitoring system should be integrated with the elevator’s structure, constantly measuring its response through sensors. The prohibiting costs of professional monitoring equipment and DAQ systems have led researchers to the development of customized, low-budget solutions, using commercial sensors and processors to build standalone DAQ systems for such applications [23]. Due to the low quality of such equipment, however, the sampled data quality is compromised, mainly due to variations in the sampling rate caused by fluctuations of the power transferred between components, thus leading to data loss. As such, a cost-effective and robust solution has yet to be provided for such applications. Lastly, it is important to mention that, while off-the-shelf monitoring systems offer a designated, tested, and validated Graphical User Interface (GUI), customized solutions usually include a very basic environment, which may not be comprehensive enough for maintenance personnel to interpret.

Aiming to mitigate the abovementioned issues, a novel computational framework is developed in this work, for SHM of an elevator system of a high-rise test tower. The developed framework aims to provide anomaly detection as well as damage classification in the elevator system, through an ensemble of DL algorithms, namely autoencoders and CNNs, trained on healthy state experimental data and damaged state data provided by high-fidelity MBD simulations. A DAQ system is installed on the elevator cabin, allowing for online monitoring and measurement extraction, while a designated interface is also developed to aid in the task of DAQ. The trained autoencoder and CNN ensembles are integrated into a novel DL-SHM framework and are employed for damage detection and classification of early-stage faults in the elevator’s door subsystem. The developed DL-SHM framework is proven robust in damage detection and classification against multiple datasets, yielding promising results and hinting at the value of the proposed simulation and DL-based SHM framework. Based on the above, the contributions of the present work can be summarized as follows:

(1) A novel damage detection and identification framework is developed using a combination of Autoencoders and CNNs, trained with healthy state vibration measurements and high-quality simulation data,
(2) Optimal MBD models reflecting multiple health states of a complete elevator system are developed and compared against their physical counterparts, validating the modeling of the various fault mechanisms in the system.
(3) A methodology for data generation using numerical simulations is presented, aiming to mitigate the problem of data scarcity which plagues the majority of real-life applications.
(4) A dedicated data acquisition system and software is developed, allowing for online data mining and condition monitoring of the elevator system.

## 2. Experimental System and Measurements

### 2.1. Experimental Elevator and Tower System

**Figure 1:**
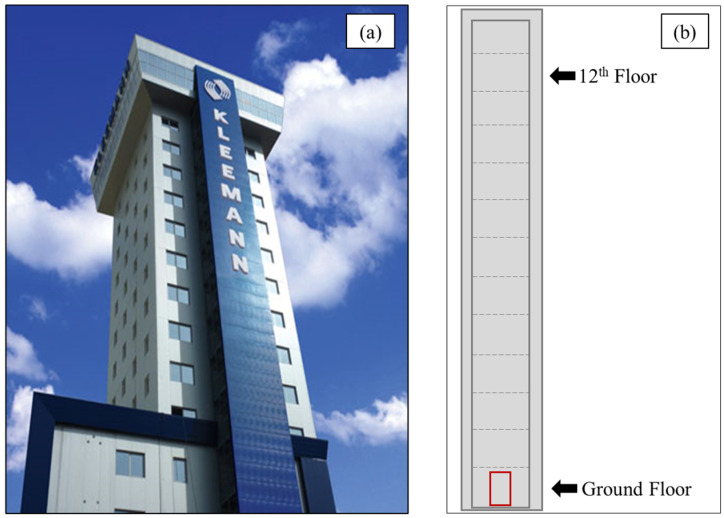
Kleemann test tower (**a**) and experiment floors (**b**).

In this work, the DL-based SHM framework was developed and validated on a high-rise test tower structure, specifically designed for developing and testing elevator systems by Kleemann Lifts (Figure 1a). The tower is located in the industrial area of Kilkis, in Central Macedonia, Greece. The tower consists of a total of 13 floors, with the 13th floor being the control room, while only floors 1 (ground) to 12 are available for elevator testing (Figure 1b).

**Figure 2:**
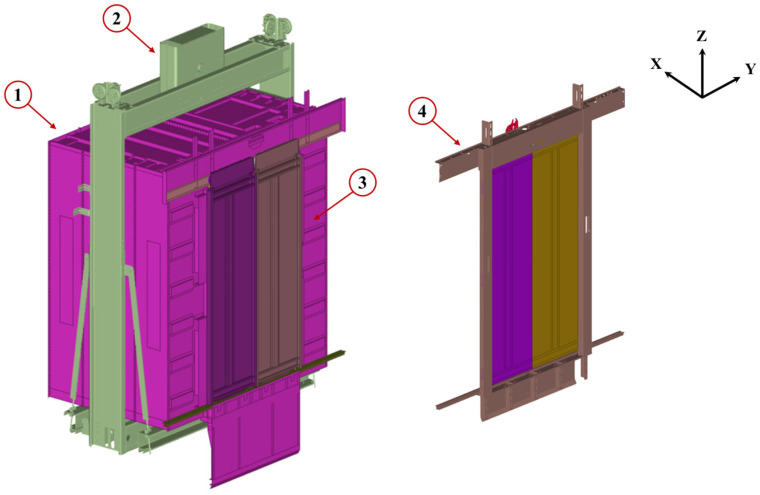
Experimental elevator and subsystems. Subsystems (1)–(3) denote the elevator cabin, chassis, and doors and subsystem (4) denotes the building floor door.

**Figure 3:**
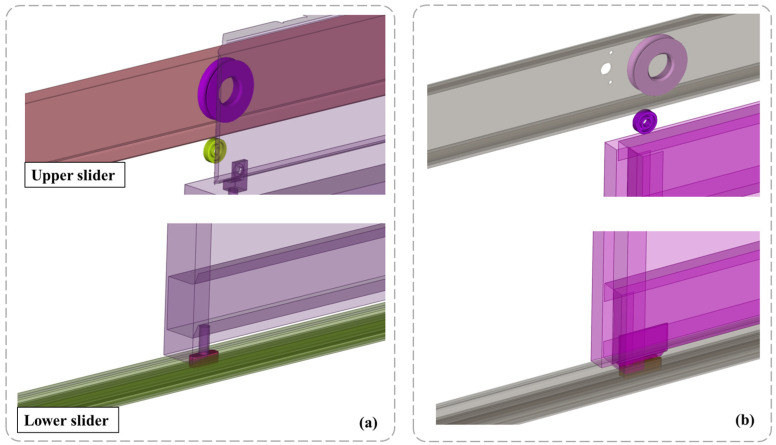
Elevator cabin (**a**) and floor (**b**) door sliding mechanisms.

The experimental elevator system used in this work is shown in Figure 2. The cabin system can be subdivided into three major subsystems, namely, the elevator cabin (1), the chassis (2), and the cabin doors and frame (3). In this study, the sliding door mechanism at the building floors was also considered and thus, the floor door subsystem (4) is also included in the analysis. The cabin consists primarily of steel, with its electronics placed in a box on the outside of the cabin roof, where a suitable place is provided for securing the required DAQ equipment. The cabin doors operate through a sliding mechanism, moving through wheels, bearings, and sliders within the cabin’s guiding rails, as shown in Figure 3a. A similar mechanism is also present in the floor door subsystem, shown in Figure 3b.

### 2.2. SHM Data and DAQ Equipment

In this work, SHM is achieved by measuring and analyzing vibration data, namely accelerations, measured at the center of the roof of the elevator cabin. Vibration-based damage detection is perhaps the most common damage detection method, with ample applications for various types of structures and machinery [24,25,26,27,28,29], as measurements can be extracted even during the operation of a system, by placing sensors on locations available on its structure. Fault-related features that are present in a system’s acceleration response have also proven useful in damage detection and classification in similar systems in the author’s previous works [17].

**Figure 4:**
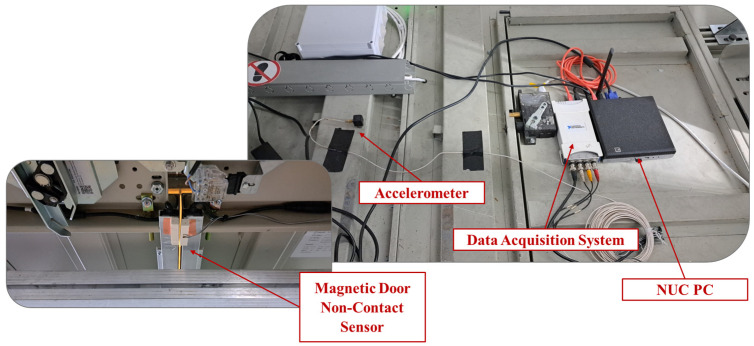
DAQ system—Acceleration and proximity sensor placement.

Aiming to minimize the number of components in the DAQ system used in this work, a single triaxial piezoelectric acceleration sensor was used for measuring the elevator’s vibration response and sampling the required SHM data. The sensor was coupled with an A/D card for signal conversion, while a NUC PC was used for data storage as well as coordination of the sampling process. Last, a magnetic proximity sensor was placed on the cabin doors in order to keep track of the cabin’s current state (open/closed). The abovementioned DAQ system is shown in Figure 4.

**Figure 5:**
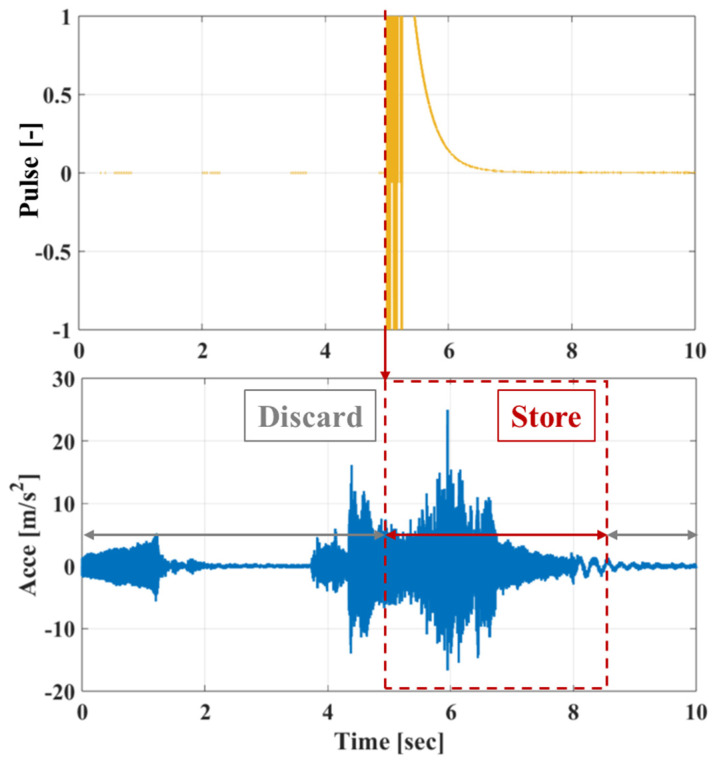
DAQ and measurement storage protocol—doors opening.

**Figure 6:**
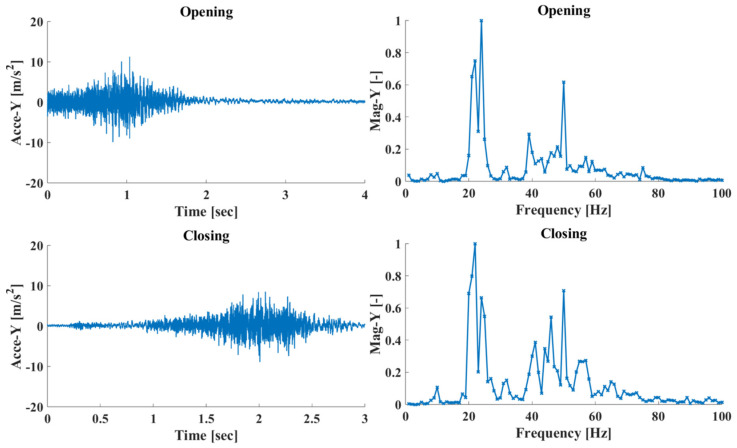
Healthy elevator acceleration response in the time (**left**) and frequency (**right**) domain.

In this work, SHM is focused on the elevator system’s door mechanisms, both for the cabin and the floor doors. As such, measurements of the system’s acceleration response were taken during the door’s opening/closing phases, where the door sliders move along their tracks. For this purpose, the magnetic proximity sensor was used as a trigger for initiating the measuring sequence, where data were stored for a short time window following the sensor’s trigger (door opening) or prior to it (door closing), as shown in Figure 5. The measured system response while opening and closing the cabin doors is shown in Figure 6, for the Y axis of the acceleration sensor, in the time and frequency domains. The data were sampled with a fixed rate of 2048 Hz, yielding a very fine resolution for the phenomena in the frequencies of interest, which are below 100 Hz. It should be noted that while Figure 6 shows both time and frequency domain data, the task of SHM was performed using only frequency domain data, for axes X and Y (horizontal plane) while opening and closing the elevator doors, resulting in data samples of size 4 × 100.

**Figure 7:**
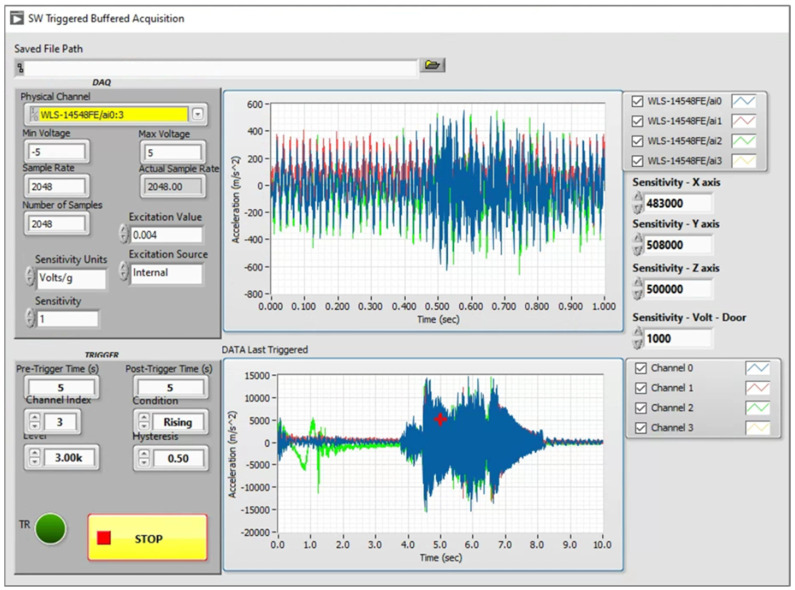
DAQ system GUI.

For the task of DAQ as well as for being able to visualize the system’s vibration response from the measuring equipment in real time, dedicated software and GUI were developed using the LabVIEW Software (2021). The software was installed in the NUC PC and was initialized once in order to start the DAQ process. The GUI during the operation of the DAQ system is shown in Figure 7 and, as shown, the user has direct access to all three acceleration channels corresponding to the X, Y, and Z axes as well as the pulse signal from the proximity sensor.

### 2.3. Elevator Door Damage Cases

As previously stated, the SHM framework developed in this work aims for the detection and classification of damages in the elevator’s door subsystem. The door subsystem was selected as the candidate for damage for two main reasons: (a) damages in the elevator doors do not compromise the rest of the system which runs the risk of destroying the elevator and (b) damages to the elevator doors are usually subtle and thus hard to detect, providing a challenge for the DL-based SHM algorithms. Damages were artificially induced to the cabin door rails as well as the 12th-floor door rails and in different severity magnitudes, leading to a total of six (6) damage cases, as stated in Table 1.

**Figure 8:**
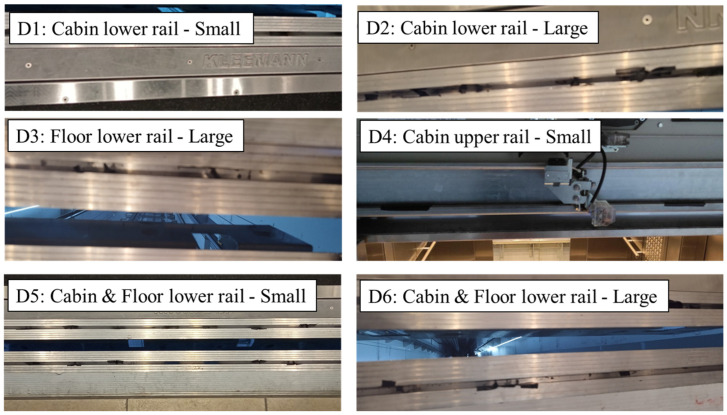
Artificial damage cases on cabin and floor door rails.

The obstacles and bumps on the elevator door rails were simulated by placing small pieces of semi-hard plastic tapes inside the rail grooves, with the different severity magnitudes being achieved through denser or more tapes along the track. The damages induced to the elevator system are shown in Figure 8.

## 3. MBD Model and Numerical Simulations

### 3.1. MBD Modeling

**Figure 9:**
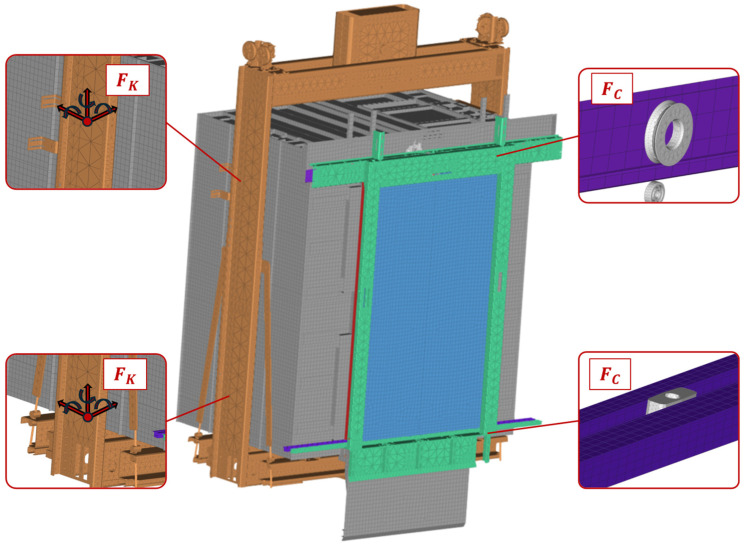
Elevator system MBD model—healthy state.

Considering that damaged state data are not usually available prior to the initial appearance of a defect in a mechanical system, for the DL-based SHM framework to be functional from the initial deployment of an elevator, training data are generated through MBD models of the physical system. The MBD model of the system in its healthy state is shown in Figure 9. The model consists of a total of 37 bodies, including the ground, cabin, rails, doors, frames, chassis, wheels, and sliders. The cabin and doors are modeled as flexible bodies as their deformations greatly affect the modal response of the system. The elevator cabin is connected to the chassis through a stiffness and damper force component, acting in all directions, similar to a bolted joint. Lastly, a contact force component is defined between the wheels, bearings and sliders, and the door rails. This contact mechanism is a key component of the MBD model, as its accurate modeling directly affects the signal produced during the operation of the sliding doors in their healthy and damaged states.

The contact force model used in the sliding door mechanism can be formulated as:

(1) $$F_{C}=F_{n}+F_{r}$$

where $F_{n},\;F_{r}$ is the contact normal and friction force, respectively. The normal force can be estimated through the common impact model as:

(2) $$F_{n}=K\delta^{n}+step\left(\delta ,0,\;0,\;C,\delta_{max}\right)\dot{\delta }$$

where K denotes the contact stiffness, δ is the penetration depth between the contacting bodies, and n is an exponent for modeling the nonlinearity of the contact. The damping component of the normal force is estimated through a step function, where the damping ranges from 0 to C as the penetration depth between the contacting bodies reaches its maximum allowable value. The friction force is then estimated as a fraction of the normal force as:

(3) $$F_{r}=\left\{\begin{array}{c} \mu_{s}F_{n},\;\;u<0\\ \mu_{d}F_{n},\;\;u\geq 0 \end{array}\right.$$

where $\mu_{s},\;\;\mu_{d}$ denote the static and dynamic friction coefficients and u is the relative velocity between the contacting bodies in the direction perpendicular to the normal force.

Acceleration data are extracted from the model from the same location on the cabin body as in the physical elevator system, and the results are sampled from the model with a rate of 2048 Hz, that is, they are printed at an interval of 1/2048 s, matching that of the physical measurements. The simulation step, however, has to be considerably smaller, as the contact phenomena in the simulation can lead to numerical instabilities and result in simulation failure due to singularities. Thus, the simulation step was an order of magnitude smaller, that is 1/20,480 s.

The model parameters for the contact force acting on the door mechanism as well as the various stiffness and damping components in the joints between the cabin and the chassis were fine-tuned based on the author’s findings from previous works such as [18]. and especially [17], where a similar system was examined, as well as through optimization by comparison between the simulated and measured healthy system responses, which are generally available since the initial deployment of a system, using the Covariance Matrix Adaptation Evolution Strategy (CMAES) [15]. This fine-tuning process is also described in greater detail in the authors’ previous works [15,17,18]. The final model parameters are shown in Table 2. In the table, the contact force-related parameters are those stated in Equations (2) and (3), while parameters KT, CT,KR, and CR are the stiffness and damping components of the joints between the elevator cabin and chassis. It should be noted that, for simplicity as well as due to the similarity of materials and geometries, these contact parameters are common both for the cabin and floor door subsystems.

### 3.2. Damaged State MBD Models

**Figure 10:**
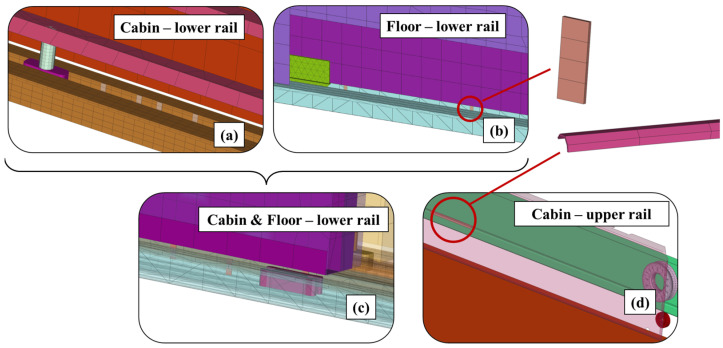
Elevator system MBD model—damaged states: (**a**) Cabin lower rail, (**b**) Floor lower rail, (**c**) Cabin and Floor lower rail, and (**d**) Cabin upper rail.

Training the DL algorithms for damage classification requires labeled training data for each of the six damaged system cases that were mentioned in Section 2.3. Using the fine-tuned MBD model of the elevator system, six damaged state models were developed by introducing the fault mechanisms to the healthy model. For each of the damage cases, additional geometries, simulating the obstacle-like tapes that were placed on the physical system, were added to the elevator door rails, as shown in Figure 10. Figure 10a shows the cabin door lower rail damage. Figure 10b shows the floor door lower rail damage. Figure 10c displays the combined cabin and floor door damage, and Figure 10d shows the cabin upper rail fault. The figures correspond to damaged states D1 and D3–D5, while the corresponding increased severity magnitude cases, namely D2 and D6 are omitted for the sake of brevity, as they are similar to cases D1 and D5 (increased number of obstacles).

Considering that the introduction of damages to the system requires the addition of bodies with different properties than those of the steel guiding rail, the damaged state model’s definition requires additional parameters, relative to the contact forces between the obstacle-like tapes and the door wheels and sliders. Given that the obstacles are of a softer material, the additional contact parameters for the damaged state models are shown in Table 3.

## 4. DL SHM Algorithms

Due to the complexity of the application as well as the fact that the elevator system is in an industrial environment, the task of damage detection and classification for subsequent SHM is considerably more demanding compared to the authors’ previous works [17,18]. This increased complexity stems from two main reasons: first, the measured vibration signal from the elevator system is contaminated by much more noise due to the industrial environment and, second, there are multiple sources of uncertainty, such as fluctuations in temperature throughout the day, the humidity in the test tower, etc., which the MBD model does not take into account, thus leading to an expected model error. Due to this, as well as the fact that damage classification through the DL algorithms is entirely dependent on the fidelity of the simulation data, the task of SHM was divided into two subtasks, that is, that of damage detection and subsequent classification. Damage detection is achieved by utilizing the healthy state experimental measurements to train an ensemble of autoencoders for predictions on the system’s response while classification is performed by first training an ensemble of CNNs using the high-fidelity simulation data and subsequently using it to discern the different damage cases. The overall DL-based SHM framework is explained in the following subsections, as well as in Algorithm 1.
**Algorithm** **1.** DL-SHM algorithmStep 1. *Load measured dataset* $X_{M}$ Step 2. *Create* $X_{in}$ *by preprocessing* $X_{M}$ Step 3. *Pass* $X_{in}$ *through the autoencoder ensemble* Step 4. *Calculate error* $e_{r}$*= err_fun(*$X_{out},X_{in}$*)* Step 5. *If* $e_{r}$ *within specified limits:*     *Label data as Healthy*   *else:*     *Label* $X_{in}$ *as Damaged*     *Pass* $X_{in}$ *through the CNN ensemble*     *Classify* $X_{in}$

### 4.1. Damage Detection Through Autoencoders

Autoencoders are DL algorithms that are primarily used on data for feature extraction, dimensionality reduction, or compression [30,31] and they are comprised of two ANNs, namely, an encoder and a decoder. The encoder is used to reduce the size of the input data by extracting the most valuable information and compressing it in the latent space while the decoder is a network used to reconstruct the original data from the latent space features. When training an autoencoder model, its parameters are updated by aiming to minimize a loss function, such as the Mean Squared Error (MSE), Root Mean Squared Error (RMSE), Mean Absolute Error (MAE), etc., between the original and reconstructed signal.

The most common architecture of an autoencoder is that of multiple fully connected dense layers. Each of these layers may contain one or more neurons which process data using a function of the following form:

(4) $$h_{j}=f_{a}\left(w_{i,j}x_{i}+b_{j}\right)$$

where w and b denote the weight and bias coefficients and x is the input data. The subscripts i,j denote the data point and neuron respectively. Lastly, $f_{a}$ denotes the activation function of the layer, which is common between all the layer’s neurons and, while it can be omitted, is usually added to enhance the network’s ability to capture nonlinear phenomena with greater accuracy.

**Figure 11:**
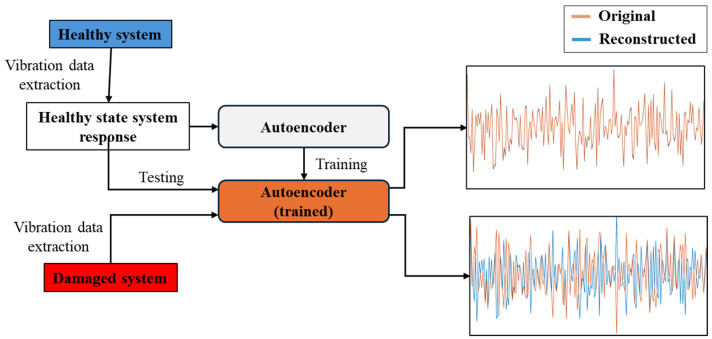
Autoencoder-based damage detection framework.

In this work, autoencoders are employed for damage detection in the following manner: when trained on data from a specific health state, an autoencoder will only be able to accurately encode and decode datasets that share characteristics with its training data. Furthermore, the more a data sample deviates from those used in the autoencoder’s training, the greater the error between the true and reconstructed signals. This property of autoencoders deems them capable of damage detection, as an autoencoder trained on healthy state data will not be able to accurately reconstruct a system’s response if the presence of damage in its structure has altered its response, thus leading to an increased reconstruction error. This autoencoder-based damage detection framework is described in Figure 11.

**Figure 12:**
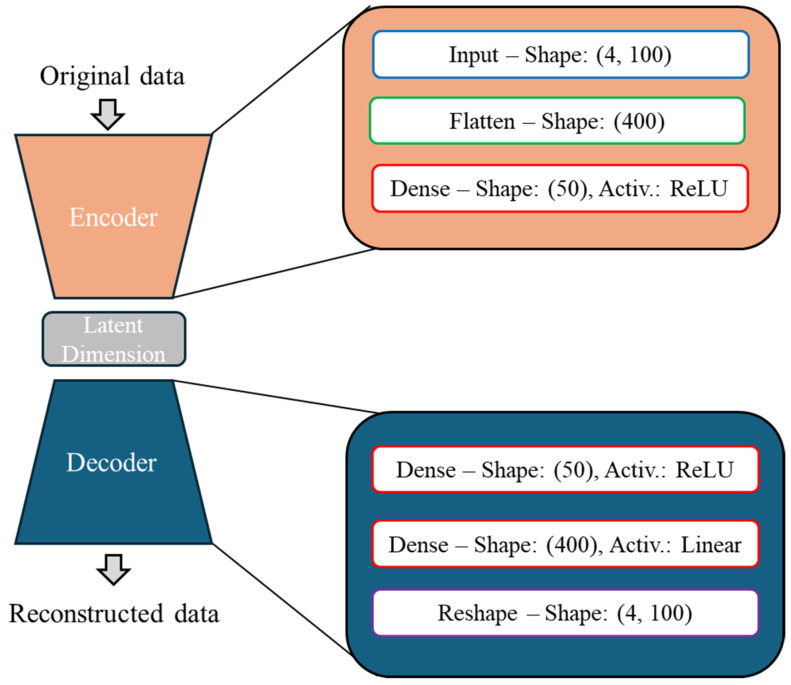
Damage detection autoencoder architecture.

The autoencoder developed for damage detection in this work is comprised of the input and output layers, three fully connected dense layers, and a flattening and a reshaping layer, as shown in Figure 12. The flattening layer is used to ensure that the data passing through the autoencoder are one-dimensional while the reshape layer is used at the output, to produce a dataset of a shape identical to that of the original data. The data samples are constructed by using the system’s acceleration response in the frequency domain, for up to 100 Hz, on axes X and Y of the accelerometer and for both the opening and closing procedures of the doors, thus yielding a shape of 4 × 100. The Adam optimizer was used for training the networks, while the loss function used was the commonly known MSE, formulated as:

(5) $${loss}_{a}=\frac{1}{N}\overset{N}{\underset{i=1}{\sum }}{\left(y_{i}-{\hat{y}}_{i}\right)}^{2}$$

where N is the number of data points, and $y,\;\;\hat{y}$ denote the system’s original and reconstructed response, respectively.

### 4.2. CNN-Based Health State Classification

While initially developed for tasks such as image recognition, CNNs have proven to be efficient classifiers in signal-related applications as well. This is primarily due to their powerful feature extraction capabilities through the application of multiple filters on the input data, as well as the convolution process which is perfect for capturing nonlinear relationships in data. Convolutional layers operate in a manner similar to that of dense layers, through a function of the form:

(6) $$g={\left(x*k\right)}_{i}=\underset{\tau }{\sum }x_{i-\tau }\;k_{\tau },i\mathrm{takes}\mathrm{all}\mathrm{valid}\mathrm{values}$$

**Figure 13:**
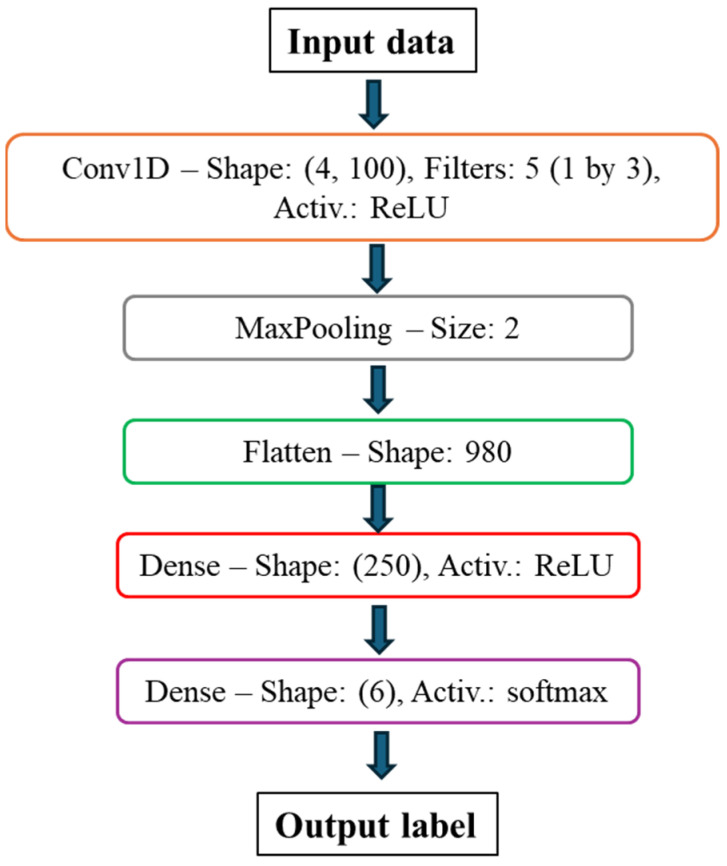
Health state classification CNN architecture.

Here, a filter or kernel k is applied on the input data x, over a number of rows τ. The ∗ operator denotes the commonly known convolution operation. When used as classifiers, the output of a CNN is a label, usually encoded in the form of zeros and ones, denoting the class of a specific dataset. For multiclass classification problems such as the task of health state classification shown in this work, the output layer of the CNN uses the softmax activation function, which is formulated as:

(7) $$\sigma {\left(x\right)}_{i}=\frac{e^{x_{i}^{cl}}}{\sum_{j=1}^{N_{cl}}e^{x_{j}^{cl}}}\;\;\;\;,\;\left(i=1,\;2,\ldots N_{cl}\right)$$

where, x denotes the input to the classification layer lc containing a number of neurons $N_{cl}$, equal to the number of desired classes. The architecture of the CNN used in this work can be seen in Figure 13. The pooling layer contained in the network is used to reduce the features in the data, only maintaining the most dominant ones, while aiding in avoiding the potential overfitting of the model to the training data. The network is trained using the Adam optimizer, aiming to minimize the categorical cross-entropy loss function, which is formulated as:

(8) $$CE=-\overset{N_{d}}{\underset{i=1}{\sum }}Y_{i}\ln\left({\hat{Y}}_{i}\right)$$

where, $Y_{i}$ is the true label of the input data, ${\hat{Y}}_{i}$ is the network’s prediction, and $N_{d}$ is the number of input datasets. The data samples for training the CNN are formulated in the same manner as in the case of the autoencoder model.

## 5. Results

### 5.1. Experimental and Simulated Response Data

In this section, a comparison between the measured and simulated system responses is shown in order to display the level of fidelity of the MBD models. It should be noted that, while healthy state data are generally considered available in every application where sensors are placed on a system, the same does not hold true for the damaged state data. Similarly, the comparison between the experimental and simulated healthy system response shown in this section was also used as a means to fine-tune the healthy state MBD model and assess its accuracy. As far as the damaged state response data are concerned, while Section 5.1.2 does show a comparison between the experimental and simulated system responses to provide some insight to the reader, these measurement data were not considered available during the training of the CNNs as, in a real-life scenario, it would most likely be unavailable. In this work, the damaged state measurement data were only used for the final validation of the proposed DL-based SHM methodology.

#### 5.1.1. Healthy State Response Data

**Figure 14:**
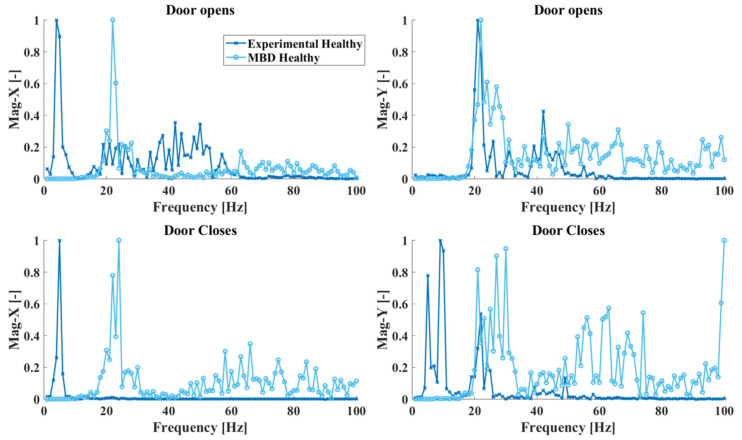
Healthy state experimental and MBD model system response in the frequency domain.

The successful assessment of the system’s health state based on the CNN ensemble is entirely dependent on the accuracy of the simulation data and their agreement with the corresponding experimental measurements. Since for the initial deployment of the DL-SHM framework, no physical measurement data are considered available for the damaged states of the system, a comparison can only be made between the healthy state experimental and simulated system responses. The measured and simulated responses for the system in its healthy state are shown in Figure 14. As shown in the figure, while the two responses do not match perfectly, the MBD model’s response contains certain dominant features that are also present in the experimental measurements, such as the peaks around 20 and 40 Hz. A certain degree of error is expected as the system at hand is quite complex, and the experiments were not performed under laboratory (controlled) conditions.

#### 5.1.2. Damaged State Response Data

**Figure 15:**
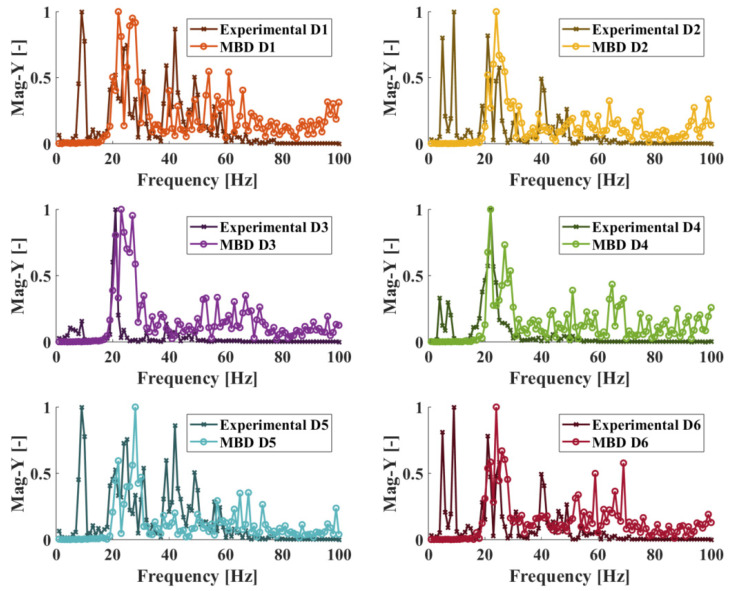
Comparison between the experimental and MBD model frequency response data for damage cases 1–6 at Y axis, doors opening.

The faulty state models of the elevator system are constructed based on the healthy state one and as such, their responses in the frequency domain are expected to display similar traits as to the healthy system’s simulated response. The main differences that are expected in the responses are those related to features appearing due to the presence of the fault mechanisms in the system. It should be noted that for the CNN classifiers to be able to generalize their predictions on physical measurements, the damaged state simulated responses should display characteristics similar to those contained in the physical system’s response. The experimental and MBD model system responses for all damaged states are shown in Figure 15. The results are only shown for the Y axis and during the door opening process for the sake of brevity, as similar results can be observed for the X axis and during the closing of the doors. As shown in the figure, while the damaged state model responses do contain certain features that are also present in the physical system response, they do not provide a perfect match. Still, should the damage-related features be contained in the data, the CNN classifiers will still be able to achieve an acceptable level of accuracy when making health state predictions.

It should be noted that, at this point in the process, there is no means of assessing the expected accuracy of the CNNs beforehand since, as previously stated, measurements of the damaged state data are not usually available in real-life scenarios. Additionally, improving the damaged state MBD models is virtually impossible, as there is no baseline for comparison, as was done with the healthy state MBD model. The final assessment of the CNN-based damage classification can only be performed after the appearance of a specific damage case in the physical system and its successful or unsuccessful detection.

### 5.2. DL-Based SHM Algorithm Training and Results

As previously stated, the task of SHM is divided into damage detection and classification, performed by an ensemble of autoencoders and CNNs respectively. The autoencoders are trained on healthy state measurement data while the CNNs are trained on damaged state data from the corresponding MBD models. For this purpose, a total of 4200 data samples were collected from the physical elevator system, 2400 of which correspond to the system’s response during operation in its healthy state while the rest were extracted from the system during the presence of faults (roughly 300 samples for each damaged state) and were used for the final validation of the proposed SHM framework. Last, from the damaged state MBD models of the system, 2000 datasets were created for each health state, resulting in a training set of 12000 samples for the CNN ensemble.

For the autoencoder ensemble, 70% of the healthy state data samples were used as training data, while the rest were used alongside the 1800 damaged state samples during the validation of the model. These data samples were used to train an ensemble of ten (10) autoencoders for the task of damage detection, by estimating the average autoencoding error for the healthy state data and classifying the samples, which results in a higher reconstruction error for damaged state data.

**Figure 16:**
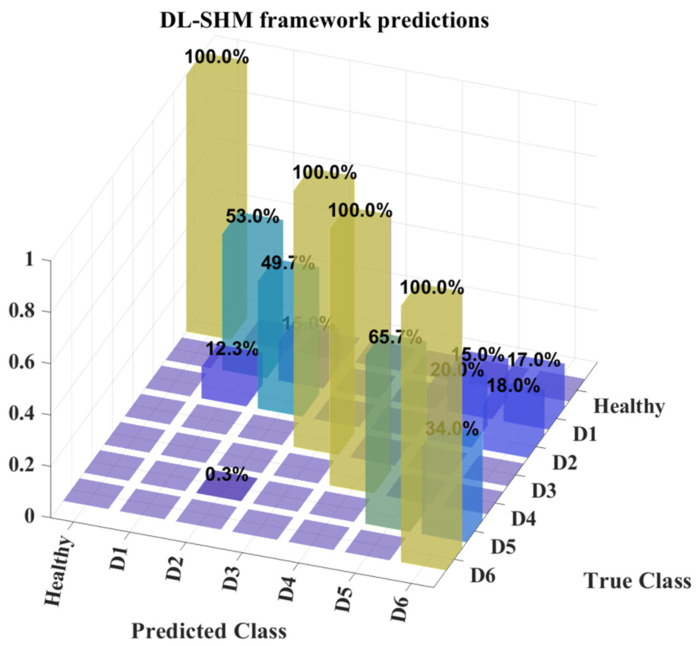
DL-SHM framework prediction results—Confusion matrix.

The CNN ensemble constructed consists of a total of ten (10) CNNs, trained on 85% of the simulated damaged state datasets while the other 15% was used for validation. The commonly used 70% to 30% analogy in the training and validation data was not maintained here, as the trained ensemble should ultimately be validated on the experimental data. The 15% validation set was used here mainly to ensure that the trained ensemble is not biased toward any of the classes in the training data. The prediction results of the DL-SHM framework’s implementation on the elevator system are shown in the confusion matrix of Figure 16.

The confusion matrix of Figure 16 presents the overall predictions from the DL-SHM framework, that is, the autoencoder as well as the CNN ensemble. As shown, there is a 100% accuracy in the predictions between the healthy and damaged states of the system, which makes sense, given that the autoencoders were trained based on the physical measurements of the system and no model error is present. The accuracy of the classification results, however, is considerably lower, at 78.07%. This indicates that the fidelity of the damaged state training data was not high enough to allow for accurate generalization of the predictions on physical measurement data. It is noteworthy however, that, while there is considerable error in the CNN ensemble predictions, this error emanates from misclassification between the D1, D2, D5, and D6 states, which correspond to damages in the cabin doors and combined damages in the floor and cabin doors. This may indicate that these datasets hold similar characteristics, given that the fault mechanisms in the system are quite similar.

Additionally, it is worth noting that there are no misclassifications regarding states D3 and D4, where the fault mechanisms affect the floor doors and the cabin top rail respectively. This is further indication that the CNN ensemble is capable of discerning these damaged states from the rest, as the fault mechanisms are quite different and their response contains different features in the frequency domain.

## 6. Discussion

At this point, it is worth breaking down further the predictions results shown in Figure 16 in order to assess the value of the proposed framework. It is worth reviewing the prediction results in two different aspects.

First, it is important to review the results from an SHM and maintenance-related aspect. Even though the DL algorithms misclassify between four out of the six damaged states, the fact that the D3 case, which is related to the specific floor where a defect is present, is accurately classified, means that maintenance and repair can be performed in an optimized manner, given that focus can be placed on a specific component of the elevator system. This is especially important when one considers that in high-rise buildings, where the number of floors exceeds the 13 floors that are present in the test tower of the current study, being able to locate the precise floor where a defect is present can help speed up the repair process, as maintenance can target that floor without the need to examine the entire building to locate the defective elevator component.

**Figure 17:**
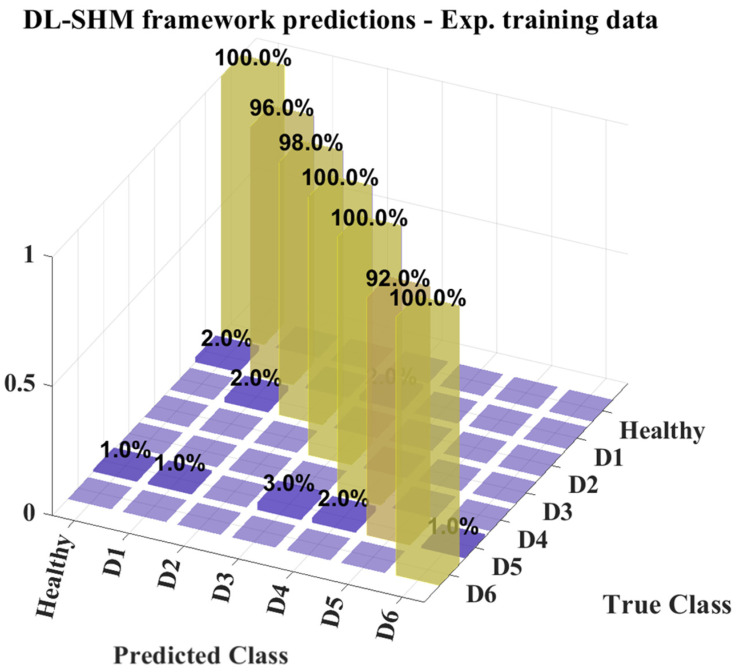
DL-SHM framework prediction results after additional training with physical measurements—Confusion matrix.

Second, it is worth noting that, due to the lack of damaged state data from the physical system, there is no means of developing a DL classifier that is ready to use from the initial deployment of the elevator system. With this in mind, the 78.07% classification accuracy achieved from simulated data is a considerable advantage over damage detection alone, which could have been achieved through the use of healthy state data and autoencoders. This accuracy can be improved as the operation of the elevator system progresses, as data can be collected at all times and, if a defect is detected in the system and then classified, it can be used to further improve the CNN classifiers through additional training. This can be seen in the prediction results, shown in the confusion matrix of Figure 17, where an overall accuracy of more than 97% is achieved by improving the CNN ensemble using experimental data from each health state.

## 7. Conclusions

In this work, a novel DL-SHM framework based on high-fidelity MBD simulation data is developed for damage detection and classification on elevator systems. The developed framework is implemented on an elevator system in a 13-story test tower, aiming to perform damage detection and classification on six (6) faulty states of the system, regarding the elevator’s cabin and floor door mechanisms. The developed SHM framework is proven to achieve highly accurate damage detection, while classification between the different damaged states of the system is achieved with an accuracy of 78.07% when the CNN ensemble is trained on damaged state simulation data. The majority of misclassification errors appear between the data samples of cabin lower rail damages, while upper rail and floor door defects are accurately predicted, hinting to the potential of the developed framework in aiding with the task of maintenance through the localization of damages before repair. The findings of this work provide great insight as to the effects of model error in the accuracy of damage classification, while the novel SHM methodology developed provides a promising foundation for further improvement of the simulation-based DL-SHM method and its extension to other subsystems of the elevator and potentially entirely different mechanical systems.

## Figures and Tables

**Table 1:** Door damage cases characteristics.

Case	Location	Description	Magnitude
D1	Cabin lower rail	Obstacles in track	Small
D2	Cabin lower rail	Obstacles in track	Large
D3	Floor lower rail	Obstacles in track	Large
D4	Cabin upper rail	Bumps adjacent to lock in track	Small
D5	Cabin & Floor lower rails	Obstacles in track	Small
D6	Cabin & Floor lower rails	Obstacles in track	Large

**Table 2:** MBD model parameters—healthy state.

Cabin and Floor Door Contacts				Cabin and Chassis Joints			
Upper Rail		Lower Rail		Bottom		Sides	
$K_{1}$ $\left[N/m\right]$	2.245 × 10^9^	$K_{2}$ $\left[N/m\right]$	1.125 × 10^8^	$KT_{1}$ $\left[N/m\right]$	1.042 × 10^10^	$KT_{2}$ $\left[N/m\right]$	9.020 × 10^9^
$C_{1}$ $\left[Ns/m\right]$	988.35	$C_{2}$ $\left[Ns/m\right]$	1098.060	$CT_{1}$ $\left[Ns/m\right]$	888.330	$CT_{2}$ $\left[Ns/m\right]$	1102.33
$n_{1}$ $\left[-\right]$	1.953	$n_{2}$ $\left[-\right]$	1.824	$KR_{1}$ $\left[N/m\right]$	1.021 × 10^10^	$KR_{2}$ $\left[N/m\right]$	8.545 × 10^10^
$\delta_{max,1}\;\left[mm\right]$	1.800 × 10^−3^	$\delta_{max,2}\;\left[mm\right]$	1.420 × 10^−3^	$CR_{1}$ $\left[Ns/m\right]$	1040.22	$CR_{2}$ $\left[Ns/m\right]$	1000.23
$\mu_{s,1}\;\left[-\right]$	0.090	$\mu_{s,2}\;\left[-\right]$	0.310				
$\mu_{d,1}\;\left[-\right]$	0.070	$\mu_{d,2}\;\left[-\right]$	0.220				

**Table 3:** MBD model damage-related parameters.

Cabin and Floor Door Damage Contacts			
Lower Rail		Upper Rail	
$K_{1}$ $\left[N/m\right]$	1.124 × 10^7^	$K_{2}$ $\left[N/m\right]$	2.332 × 10^7^
$C_{1}$ $\left[Ns/m\right]$	800.488	$C_{2}$ $\left[Ns/m\right]$	560.020
$n_{1}$ $\left[-\right]$	1.701	$n_{2}$ $\left[-\right]$	2.010
$\delta_{max,1}\;\left[mm\right]$	1.800 × 10^−3^	$\delta_{max,2}\;\left[mm\right]$	1.520 × 10^−3^
$\mu_{s,1}\;\left[-\right]$	0.351	$\mu_{s,2}\;\left[-\right]$	0.351
$\mu_{d,1}\;\left[-\right]$	0.243	$\mu_{d,2}\;\left[-\right]$	0.243

## Data Availability

All data and materials used in this work may be shared upon request (email to the corresponding author).
